# Oral Administration of Nanopeptide CMCS-20H Conspicuously Boosts Immunity and Precautionary Effect Against Bacterial Infection in Fish

**DOI:** 10.3389/fimmu.2021.811616

**Published:** 2022-01-11

**Authors:** Xingchen Huo, Zhensheng Wang, Xun Xiao, Chunrong Yang, Jianguo Su

**Affiliations:** ^1^ Department of Aquatic Animal Medicine, College of Fisheries, Huazhong Agricultural University, Wuhan, China; ^2^ Laboratory for Marine Biology and Biotechnology, Pilot National Laboratory for Marine Science and Technology, Qingdao, China; ^3^ Hubei Hongshan Laboratory, Engineering Research Center of Green Development for Conventional Aquatic Biological Industry in the Yangtze River Economic Belt, Ministry of Education, Wuhan, China; ^4^ College of Veterinary Medicine, Huazhong Agricultural University, Wuhan, China

**Keywords:** CMCS-20H nanoparticles, bacterial disease, immune response, antimicrobial peptide, immunopotentiator

## Abstract

Massive mortalities caused by bacterial infections in intensive aquaculture result in serious economic losses. In this study, a novel antimicrobial peptide gcIFN-20H was efficiently expressed in *Pichia pastoris* (GS115) and loaded on carboxylmethyl chitosan (CMCS) to prepare CMCS-20H nanoparticles. Through physical characterization assays (TEM, DLS, BCA, and Raman) and biological activity tests (antimicrobial activity and cytotoxicity), CMCS-20H nanopeptide was verified to be spherical nanoparticles with sustained release, antimicrobial activity, and negligible toxicity. CMCS-20H nanoparticles are more resistant to intestinal degradation than unloaded gcIFN-20H by indirect immunofluorescence assay. Oral administration was then carried out for 42 days. Complement C3 content, lysozyme, and total superoxide dismutase activities are highest in CMCS-20H group by serum biochemistry index assays. After challenge with *Aeromonas hydrophila*, the survival rate in CMCS-20H group is highest (46%), which is 64% higher than the control group (28%). Meanwhile, the tissue bacterial loads (intestine, spleen, head kidney, trunk kidney, hepatopancreas, muscle, and blood) in the CMCS-20H group are significantly lower than other groups. By PAS staining analysis, the number of intestinal villi goblet cells and the thickness of mucin in the CMCS-20H group obviously increased. CMCS-20H effectively enhances mRNA expressions of some important immune genes (IL-1β, IL-6, TNF-α, IL-2, IFN-γ2, and IgM). The minimal tissue lesions (Intestine, spleen, and trunk kidney) were seen in the CMCS-20H group by histopathological examination. 16S rRNA sequencing showed that oral CMCS-20H maintains the intestinal microbiome homeostasis in bacterial infection. The results indicate that the novel nanopeptide CMCS-20H as the immunopotentiator can remarkably boost fish immunity and precautionary effect by oral administration and address the theoretical mechanisms and insights into the promising application prospect in aquaculture.

## Highlights

1. CMCS-20H nanoparticles have the effects of slow release and prevent enzymatic hydrolysis.
2. Oral CMCS-20H improves the innate immunity against bacterial disease.
3. CMCS-20H shows excellent immune responses and highest survival rate.
4. CMCS-20H remarkably improves survival rate and mucin thickness in bacterial infection.
5. CMCS-20H maintains intestinal microbiome homeostasis in bacterial infection.5.

## 1 Introduction

Bacterial infections cause massive mortalities in intensive aquaculture, leading to serious economic losses (1, 2). The bacterial diseases of aquaculture animals are often prevented and treated by chemotherapeutants. However, this method may result in many problems, such as the production of drug-resistant bacteria, decrease of fish immune functions, and drug residues which could further affect human health (3, 4). Bacterial infections can also be prevented by vaccination (5, 6). However, commercial vaccines only prevent specific pathogens, and numerous vaccines also increase the costs (7, 8). Facing the severe bacterial diseases in aquaculture, it is urgent to develop ideal antimicrobial agents.

Immunopotentiators, based on recent successes in both laboratory and clinic, are considered a viable option for controlling bacterial diseases (9, 10). Some antimicrobial peptides (AMPs) have good immune regulation function, so they can also be used as immunopotentiators. AMPs are short peptides with cationic and amphipathic characteristics, and they widely exist from microorganisms to humans in various life forms (11). The immunomodulatory activity of AMPs mostly depends on its influence on the function of innate immune cells and mediators (12). In addition, AMPs (such as lactoferrin, polypeptide S100, LL-37, cecropin A) have the advantages of broad-spectrum antibacterial activity, small bioconcentration, and less possibility to cause bacterial resistance (13, 14). Therefore, the researches on immune enhancement of AMPs have also become a hotspot in recent years. The efficacy of AMPs is easily weakened once it enters the complex physiological environment. The bioavailability of AMPs is greatly reduced by biological digestion, thus reducing the effect of AMPs (15, 16). It is a feasible method to utilize carboxymethylation carboxylmethyl chitosan (CMCS) drug loading system to load AMPs to resist environmental impact. CMCS is a chemical modification version of chitosan (CS). CS and CMCS have been widely explored based on their biodegradability, biocompatibility, and nontoxicity (17). The functional groups on the surface of CMCS can couple with AMPs to form nanoparticles which have the effect of preventing enzymatic hydrolysis (15). The CMCS nanoparticles can slowly release AMPs at specific sites (18, 19). Through the functions of anti-enzymatic hydrolysis and sustained release, CMCS drug loading system will improve the bioavailability of AMPs. The application method of oral AMPs is suitable for elevating immunity of aquatic animals (20, 21). In order to improve the applicability, the production of AMP needs to fulfil the requirements of high yield and low cost. In many expression protein systems, *Pichia pastoris* expression system is a good choice because of its eukaryotic property, efficient secretion capacity, and high-cell cultivation ability (22, 23). In addition, methanol as inducer could further lower the costs in *P. pastoris* culture under the precise regulation of AOX1 promoter (24).

*Aeromonas hydrophila* is generally considered a major pathogen in almost all the animal taxa, causing hemorrhagic septicemia and intestinal inflammation in freshwater fish (25, 26). Due to the intensive culture, the bacterial diseases usually break out in the farmed commercial fish such as grass carp (*Ctenopharyngodon idella*). The bacterial enteritis induced by *A. hydrophila* is regarded as one of the most frequently occurring diseases in grass carp culture (27). In order to prevent the diseases caused by *A. hydrophila*, the intestinal inflammation model is developed in grass carp (26). This inflammation model can be applied to test antimicrobial drugs for potential use in aquaculture.

Our previous study found that grass carp IFN1 is a highly effective AMP (28). The fifth α-helix was identified as a novel AMP (gcIFN-20) with a typical cationic AMP structure and broad spectrum of bactericidal activity (unpublished data). In this study, we expressed gcIFN-20H through yeast expression system and prepared CMCS-20H nanoparticles by CMCS loading. The precautionary efficacy and mechanisms for bacterial diseases by oral CMCS-20H nanoparticles were investigated by grass carp against *A. hydrophila* infection. The results demonstrated that CMCS-20H exhibits excellent immune enhancement effect, which lays a solid foundation for the application of novel nanopeptide CMCS-20H in aquaculture.

## 2 Materials and Methods

### 2.1 Preparation of Experimental Basic Materials

Healthy grass carp (weight = 25 ± 5 g) were obtained from a fish farm in Fanzhou, Hubei Province, China. Healthy fish were randomly divided into eight experimental groups (50/group), each tank was filled with 250 L of water, and the water temperature was kept at 25°C ± 1°C. The animals acclimatized for 4 weeks before experiments, and we fed the grass carp with commercial feed twice a day. All procedures of animal experiments were approved by the Ethical Committee on Animal Research at Huazhong Agricultural University. All the efforts were made to minimize animal suffering. CMCS (deacelation degree = 85%, CAS: 83512-85-0) was provided by Santa Cruz (Dallas, TX, USA).

The *P. pastoris* GS115 was stored at −80°C in our laboratory. GS115 was grown in yeast extract peptone dextrose (YPD) liquid medium at 28°C, 200 rpm. *C. idella* kidney (CIK) cells were cultured in M199 (Gibco, Waltham, MA, USA) medium, supplemented with 10% FBS (Gibco, USA), 100 U/ml penicillin, and 100 U/ml streptomycin and maintained at 28°C in a humidified atmosphere of 5% CO_2_ incubator. *A. hydrophila* (ATCC7966) was stored at −80°C in our laboratory. The single colony was formed by inoculating a Luria–Bertani (LB) agar plate with 80 μl of cryopreserved *A. hydrophila* and incubated at 28°C for 48 h. A bacterial colony was then selected and inoculated into the LB medium and cultured in a shaking incubator at 150 rpm for 24 h at 28°C.

### 2.2 The Process of Yeast Expression of gcIFN-20H

Yeast expression system was used to express gcIFN-20H according to a previous method (22). According to the codon usage of *P. pastoris*, gcIFN-20H DNA sequence was optimized. The lower-frequency codons were replaced with synonymous codons with a higher frequency in *P. pastoris*, and accompanied by adjusting AT-rich region for suitable G + C content. 6 × His-tag sequence was linked at the end of gcIFN-20 DNA sequence. The optimized gcIFN-20H DNA sequence (GenBank accession number: OK413875) was synthesized by Tsingke Biological Technology (Beijing, China). The optimized sequence was inserted into the pPIC9K (p9K) plasmid to obtain pPIC9K-gcIFN-20H (p9K-20H). p9K vector was the negative control vector.

The p9K-20H were linearized using *Sal* I and transformed into *P. pastoris* GS115 by electroporation (7,000 V/cm, 25 μF, ×400; Life Technologies CellPorator, Carlsbad, CA, USA). Transformants were selected on minimal dextrose (MD) medium plates (1.34% YNB, 0.00004% biotin, 2% dextrose, 1.5% agar) without histidine. Clones from the MD plates were then selected in YPD+G418 (Invitrogen, Shanghai, China) plates (1% yeast extract, 2% peptone, 2% dextrose, 2% agar, and 0.2–0.8 mg/ml G418). Recombinants bearing different copies of the target gene were inoculated into 10 ml buffered glycerol-complex medium (BMGY; 1% yeast extract, 2% peptone, 1.34% YNB, 0.00004% biotin, 1% glycerol, 100 mM potassium phosphate, pH 6.0) and incubated at 28°C for 2 days. The cells in each culture were collected by centrifugation at 4,000×*g* for 5 min and individually inoculated into 100 ml buffered minimal methanol YP medium (BMMY; same as BMGY but replacing glycerol with 0.5% methanol). A total of 1% (v/v) methanol was then added every 24 h to induce the expression of the foreign protein. The p9K was also operated according to the above experimental procedure. In addition, gcIFN-20H in the supernatant were separated and purified by His-tag Protein Purification Kit (P2226; Beyotime, Haimen, China). Protein concentrations were determined using the Bradford method.

### 2.3 Preparation of Nanopeptide CMCS-20H

#### 2.3.1 Polyacrylamide Gel Electrophoresis Analysis and Western Blot

The gcIFN-20H in the culture supernatant (10 μl) induced by methanol were analyzed on 16.5% Tris-Tricine-SDS-PAGE and Western blot (WB). The samples from shaken flask experiments were separated by Tris-Tricine-SDS-PAGE. Gels were stained by Coomassie brilliant blue R-250. Also, the gels were transferred onto the hybridization nitrocellulose (NC) filter membrane (Millipore, Burlington, MA, USA). After transfer, the membrane was blocked with 5% skim milk diluted in phosphate-buffered solution-Tween-20 (PBST, Boster, Wuhan, China) for 2 h. After washing with PBST, the blocked membrane was incubated with the mouse anti-His-tag monoclonal antibody (1:3,000, ABclonal, Woburn, MA, USA) for 2 h. Subsequently, the membrane was rewashed with PBST and incubated with a 1:2,000 dilution of horse radish peroxidase (HRP)-conjugated goat antimouse IgG antibody (Boster, Wuhan, China) for 1 h. The NC filter was washed again with TBST, subsequently stained with Clarity TM Western ECL Substrate (Bio-Rad, Hercules, CA, USA), and finally imaged by the Amersham Imager 600 (Little Chalfont). Purified proteins and control (10 μl culture supernatant of GS115 that transfected with p9K) were also detected by the above method.

#### 2.3.2 Conjugating gcIFN-20H and CMCS and Characterizing CMCS-20H

CMCS-gcIFN-20H nanoparticles (CMCS-20H) were prepared according to the previously described ionic gelation methods (18, 29). Briefly, gcIFN-20H (1 mg/ml) and CMCS (1 mg/ml) solutions were premixed for 2 h under magnetic stirring to determine the optimal concentration of gcIFN-20H, and the formed primary structure was termed as CMCS-20H nanoparticles. CMCS-20H was collected *via* ultracentrifugation (12,000 rpm, 4°C, 30 min) and was washed with sterile ultrapure water twice. The encapsulation efficiency (EE) and loading efficiency (LE) of CMCS-20H nanoparticles were determined according to the previous method (30). After centrifugation, the amount of gcIFN-20H encapsulated in CMCS nanoparticles was determined by measuring the amount of protein remaining in the supernatant by bicinchoninic acid (BCA) protein assay. The EE and LE were calculated according to formulas (1) and (2):


(1) EE=(A−B)/A×100%

(2) LE=(A−B)/C×100%

where *A*, *B*, and *C* refer to the weight of total gcIFN-20H used and gcIFN-20H nonencapsulated and gcIFN-20H-loaded CMCS, respectively (*n* = 3).

The release of gcIFN-20H from the nanoparticles was measured according to the previous description (19). In the released medium (PBS, pH = 7.4 and pH = 5.2), *in vitro* release profile of CMCS-20H was detected at 25°C for 20 h. CMCS-20H (1 mg) was placed into EP tubes with 2 ml of release medium in a shaking incubator at 100 rpm. The protein content in the supernatant was measured by BCA assay method within the specified time. The release efficiency (RE) was calculated according to formulas (3):


(3) RE=D/B×100%

where *D* and *B* refer to the weight of gcIFN-20H in supernatant and carried respectively (*n* = 3).

The formed nanoparticles were resuspended in distilled water (pH = 6.4) using a probe sonication (pulse on, 3.0 s; pulse off, 2.0 s; 1 min/cycle; power 130 W) characterization. The particle size distribution and zeta potential of CMCS-20H were determined by dynamic light scattering (DLS) using a Malvern Nano-ZS 90 laser particle size analyzer (Malvern Instruments, Royston, UK) at a detector angle of 90°, 670 nm, and temperature of 25°C. The morphology of CMCS-20H was observed by transmission electron microscopy (TEM, SPA-400, Japan).

#### 2.3.3 Cytotoxicity Test

Cytotoxicity was measured in CIK cells by 3-(4,5-dimethylthiazol-2-yl)-2,5-diphenyltetrazolium bromide (MTT) assay. The cells were seeded in a 96-well plate at a density of 10^4^ cells/well. CMCS-20H and gcIFN-20H (final concentration at 256 µg/ml) were then added into the corresponding wells. The CIK cells supplemented with fresh media and equal PBS were used as a control. After incubation for 12 and 24 h, the medium was replaced with fresh medium containing 10% MTT solution for 4 h at 28°C. The absorbance at 595 nm was measured by a microplate reader of multiwavelength measurement system. Each measurement was performed in triplicate.

#### 2.3.4 Antimicrobial Test

Antimicrobial activity is evaluated by CFU assay (31). *Escherichia coli* (ATCC 25922) and *Staphylococcus aureus* (ATCC 25923) were cultured overnight in LB medium at 37°C. The bacteria were subcultured to achieve midlogarithmic phase growth. The bacteria were washed with Tris buffer (10 mM Tris-HCl, 5 mM glucose, pH = 7.4), then diluted to a final concentration of 1 × 10^6^ CFU/ml in Tris buffer. The bacteria (1 × 10^6^ CFU) were incubated with CMCS-20H, gcIFN-20H, and CMCS (20 μg/mL) for 2 h. The mixtures were then spread onto the LB agar plates, and bacterial counts were measured at 16 h. Antibacterial experiments were tested in triplicate.

### 2.4 Indirect Immunofluorescence Analysis

Grass carp (*n* = 4) were reared in 10 L plastic aquaria that were filled with dechlorinated tap water (water temperature 25°C) and aerated. According to the LE, grass carp were respectively fed with 0.5 ml solution of CMCS-20H nanoparticles (5 mg/ml) and gcIFN-20H (1 mg/ml) by using a catheter. Grass carp were fed with PBS as control. At 6 and 12 h, intestines were excised to make frozen sections. The sections were then incubated with 5% BSA at 37°C for 1 h. After incubation, the sections were bound to primary mouse anti-His antibody (1:3,000, ABclonal, China) and secondary antibody (FITC-conjugated goat antimouse IgG, 1:200, Dia-an, China) at 37°C for 1 h, respectively. After washing three times with PBS, the sections were observed on UltraVIEW VoX 3D Live Cell Imaging System (Olympus, Shinjuku, Japan). Indirect immunofluorescence analysis of intestine was repeated four times. Fluorescence intensity was analyzed by ImageJ software.

### 2.5 Investigating the Immune Enhancement Effect of CMCS-20H *In Vivo*

#### 2.5.1 Bacterial Challenge and Survival Rate

Grass carp were divided into two kinds of experimental groups. There were 100 fish in each experimental group; 50 of them were used to measure mortality, and the remaining 50 were used for sampling. Based on previous experimental methods (18, 32), the dosage of CMCS-20H was converted into the same dose of free gcIFN-20H according to LE. The feeding situations before challenge were as follows: CMCS-20H (20 mg/kg, CMCS-20H group), gcIFN-20H (4 mg/kg, gcIFN-20H group), CMCS (16 mg/kg, CMCS group), and common fodder (control group). Each group of fish was fed twice a day for 42 days. Feeding was stopped for 1 day before the challenge and given common fodder after challenge. *A. hydrophila* was resuspended in PBS buffer (pH = 7.4). *A. hydrophila* (100 μl, 2 × 10^5^ CFU/mk) was intraperitoneally injected on D43. The mortality was monitored from D1 to D7 postchallenge.

#### 2.5.2 Tissue Bacterial Loading Assay

The tissue bacterium-loading experiment was based on the previous experimental method (33, 34). At 72 h after challenge, four fish were taken from each group, and trunk kidney, intestine, hepatopancreas, blood, muscle, spleen, and head kidney were collected and grinded by multisample tissue grinding machine (CEBO-24, Cebo, Aberdeen, UK), respectively. These tissue homogenates were used to detect the bacterial load in tissues. The homogenates were diluted using sterile PBS (pH = 7.4), spread on Rimler-Shotts (RS) medium plates (Haibo, Ningbo, China) for 12–16 h at 28°C. Colonies of *A. hydrophila* were counted by two independent investigators.

#### 2.5.3 Hematoxylin and Eosin Staining

The intestine, spleen, and trunk kidney were taken on D46 and fixed immediately in 10% neutral buffered formalin for 24 h. Tissue samples were embedded in paraffin. Four-micrometer-section samples were mounted on aminopropyl triethoxysilane-coated slides. Following the deparaffinization in xylene, sections were rehydrated, stained with hematoxylin and eosin (HE), and mounted with neutral gum, and then the images were captured. Images were captured using an Eclipse Ti-SR microscope with a DS-U3 Image-Pro system (Nikon, Minato, Japan).

#### 2.5.4 Periodic Acid-Schiff Staining

Periodic acid-Schiff (PAS) staining was used to detect goblet cells and mucus layer thickness in intestine. Intestine tissues were collected on D46 and immediately fixed in methanol-Carnoy’s fixative at 4°C for 2.5 h, and then transferred to 100% ethanol. Fixed intestinal tissues were washed with distilled water for 5 min and embedded in paraffin. Sections of 5 μm thickness were deparaffinized and stained with PAS. Images were captured using an Eclipse Ti-SR microscope with a DS-U3 Image-Pro system (Nikon, Japan). Three fields (×20) and three fields (×70) were respectively selected for goblet cell count and mucin thickness measurement. The PAS staining experiments were repeated three times.

#### 2.5.5 qRT-PCR Assay of Immune Genes

Total RNAs of head kidney tissue were isolated with RNAiso Plus kit (TaKaRa, Kusatsu, Japan), the purification and concentration were measured by a NanoDrop 2000 spectrophotometer (Thermo Scientific, Waltham, MA, USA), and the quality was evaluated using 1.5% agarose gel electrophoresis. mRNAs were reverse-transcribed into cDNAs respectively with MMLV reverse transcriptase, RNase inhibitor (Thermo Fisher Scientific, USA), hexamer random primer. The primers for qRT-PCR analyses are listed in **Supplementary Table S1**. Sequence source (GenBank): 18S rRNA (EU047719.1), TNF-α (KX094934.1), IL-1β (KX094935.1), IL-6 (MG188797.1), IL-2 (MH883895.1), IFN-γ2 (JX657683.1), and IgM (DQ417927.1). 18S rRNA was used as a reference control gene, and the relative mRNA expression levels were calculated with the CT method (2^−△△CT^).

#### 2.5.6 Serum Biochemistry Index Assay

Blood samples were collected from the caudal vein and placed for 2 h at 25°C. After centrifugation (4,500 rpm/min, 10 min) at 4°C, the serum samples were collected and stored at −80°C. The serum biochemical indexes of complement 3 (C3), lysozyme, total superoxide dismutase (TSOD), and total protein (TP) were examined by the commercial kits (Nanjing Jiancheng Bioengineering Institute, Nanjing, China). Sera of four grass carp were taken from each group for detection.

#### 2.5.7 Microbiome Analysis

We used 16S rRNA gene sequencing method to investigate the intestinal microbiota in total intestinal samples. PCR amplification of the bacterial 16S rRNA gene V3–V4 region was performed using the forward primer 338F (5′-ACTCCTACGGGAGGCAGCA-3′) and the reverse primer 806R (5′-GGACTACHVGGGTWTCTAAT-3′). Sample-specific 7-bp barcodes were incorporated into the primers for multiplex sequencing. PCR volume consists of 4 μl 5×FastPfu buffer, 2 μl dNTPs (2.5 mM), 1.0 μl each primer (5 μM), 0.2 μl FastPfu polymerase, 5.0 μl BSA, 2 μl template DNA (10 ng), and 4.8 μl ddH_2_O. Thermal cycling is composed of initial denaturation at 98°C for 5 min, followed by 25 cycles consisting of denaturation at 98°C for 30 s, annealing at 53°C for 30 s, and extension at 72°C for 45s, with a final extension of 5 min at 72°C. PCR amplicons were purified with Vazyme VAHTSTM DNA Clean Beads (Vazyme, Nanjing, China) and quantified using the Quant-iT PicoGreen dsDNA Assay Kit (Invitrogen, Waltham, MA, USA). After the individual quantification step, amplicons were pooled in equal amounts, and pair-end 2 × 250 bp sequencing was performed using the Illumina MiSeq platform with MiSeq Reagent Kit v3 at Shanghai Personal Biotechnology Co., Ltd. (Shanghai, China).

In this study, 20 samples were sequenced by Illumina MiSeq system, and a total of 93,210 high-quality sequences were obtained. On average, each sample has 44,908 reads. All the sequence reads were trimmed and assigned to each sample based on their barcodes. Sequences with high quality (length >250 bp, without the ambiguous base “N,” an average base quality score >30) were used for further analysis. Assignment of operation taxonomic units (OTUs) was completed at 97% identity using the Uclust function in web (https://www.genescloud.cn). The gut microbial community compositions at phylum and genus levels in different samples were analyzed by R software. The α-diversity metrics of intestinal microbiota, including Chao1 index, Goods coverage index, Simpson index, and Observed species index were calculated using web (http://www.genescloud.cn/analysisProcess). For the β-diversity metrics, the principal coordinate analysis (PCoA) and permutational multivariate analysis of variance (PERMANOVA) analysis were performed using OTUs for each sample through the web (https://www.genescloud.cn/analysisProcess). Kruskal-Wallis, Wilcoxon rank sum, and Dunn’s multiple comparison tests were used for hierarchy cluster analyses. Grass carp fed with common fodder and unchallenged with *A. hydrophila* were used as blank control group (BC).

### 2.6 Statistical Analysis

The results were expressed as the means ± standard deviation (SD), and all the statistical analysis were done using SPSS 26.0 package. The experimental data were subjected to the Kruskal-Wallis test, followed by Dunn’s multiple comparison (with Bonferroni adjustment) to identify the significance (*p* < 0.05). Different superscript letters in each group (a, b, and c) denote significant variations. The survival rates were analyzed by Mantel-Cox test; * denotes significant variation.

## 3 Results

### 3.1 CMCS-20H Nanoparticles Possess Antimicrobial Activity, Sustained Release, and Low Toxicity

**Figure 1:**
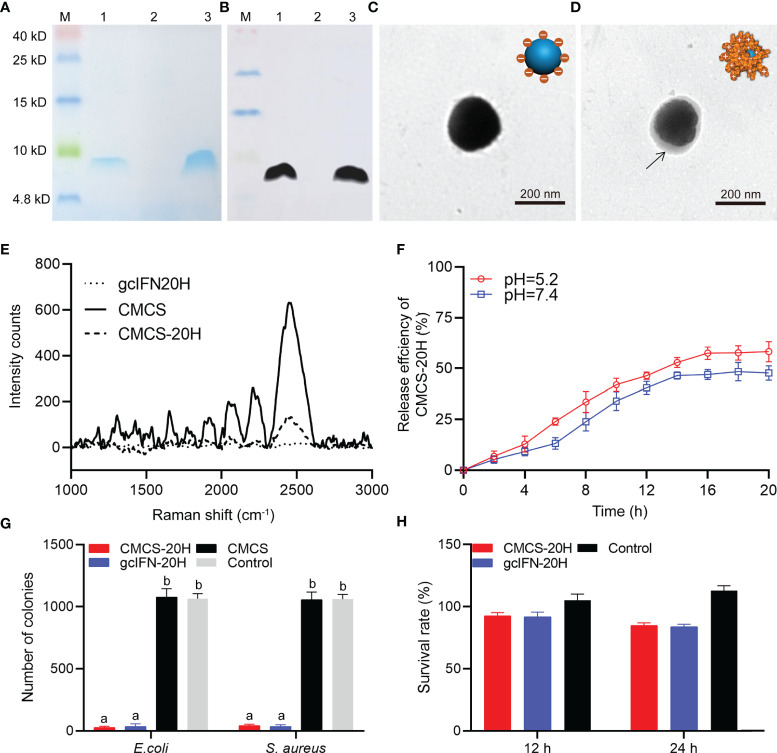
Preparation, characterization, and bioactivity of CMCS-20H nanoparticles. **(A)** SDS-PAGE and **(B)** WB analyses of gcIFN-20H. Lane M: protein marker. Lane 1: the fermentation supernatant of gcIFN-20H. Lane 2: the fermentation supernatant of blank vector as negative control; Lane 3: purified gcIFN-20H. **(C)** Transmission electron micrograph of CMCS. **(D)** Transmission electron micrograph of a CMCS-20H. The black arrow indicates the position of gcIFN-20H. **(E)** Raman spectroscopy analysis. **(F)** *In vitro* release efficiency of gcIFN-20H (plotted as a function of % cumulative release vs. time) from CMCS-20H in PBS buffer (pH = 5.2 and 7.4). **(G)** Antibacterial activity detection of CMCS-20H and gcIFN-20H. *E. coli* and *S. aure*us (1 × 10^6^ CFU) were incubated with CMCS-20H, gcIFN-20H, and CMCS (20 μg/ml) for 2 h at 37°C, respectively. The equivalent volume of Tris was used as control. **(H)** Cytotoxicity of CMCS-20H and gcIFN-20H to CIK cells. CMCS-20H and gcIFN-20H (final concentration at 256 µg/ml) were respectively incubated with CIK cells for 24 h at 28°C. PBS was employed as control. Data are presented as means ± SD (*n* = 3). Different lowercase letters in each group (a and b) denote significant variations suggested by the Kruskal-Wallis statistics followed by the Dunn’s multiple comparison (p < 0.05).

By SDS-PAGE analysis, band associated with gcIFN-20H was found in the supernatant (**Figure 1A**). In addition, the band of purified sample was confirmed as gcIFN-20H by WB using specific antibody (**Figure 1B**). These results showed that GS115 strain successfully expressed gcIFN-20H. CMCS-20H nanoparticles were prepared according to the ionic gelation methods. CMCS-20H nanoparticles form through the adsorption of negatively charged CMCS and positively charged gcIFN-20H. The unloaded CMCS was spherical with smooth surfaces in PBS solution (**Figure 1C**), and after gcIFN-20H was loaded onto the surface of CMCS, protein shadow was observed, surrounding the surface of CMCS (**Figure 1D**). Raman spectroscopy analysis indicated that numerous specific peaks were observed in the CMCS group, and no peak was observed in the gcIFN-20H group. After the surface of CMCS was covered by gcIFN-20H, the intensity of peaks in the CMCS-20H group was decreased (**Figure 1E**). By drug release analysis, gcIFN-20H was released from CMCS-20H nanoparticles in different buffers (pH = 5.2 and 7.4). Compared with that in neutral environment, CMCS-20H releases gcIFN-20H faster in acidic environment (**Figure 1F**). Compared with the control group, CMCS-20H and gcIFN-20H exhibit forceful antibacterial activity (**Figure 1G**). By MTT assay, CMCS-20H and gcIFN-20H do not show sensible cytotoxicity (**Figure 1H**).

Zeta potential of CMCS was −34.8 ± 3.3 mV. gcIFN-20H layer was added to the CMCS shell to form CMCS-20H, then the surface potential turned into +17.5 ± 2.4 mV, and the mean particle size increased to about 186 ± 13.4 nm. The polydispersity index (PDI) value of CMCS-20H and CMCS was less than 0.25. EE and LE of CMCS-20H were 26.11 ± 1.56% and 21.20 ± 1.62%, respectively (**Table 1**). These results indicate that CMCS-20H is spherical nanoparticle with features of uniform particle size, sustained release, antimicrobial activity, and low toxicity.

**Table 1:** Properties of CMCS-20H and CMCS (mean ± SD, *n* = 3).

Name	Mean particle size (nm)	PDI	Zeta potential (mV)	EE (%)	LE (%)
CMCS-20H	186 ± 13.4	0.18 ± 0.08	+17.5 ± 2.4	26.11 ± 1.56	21.20 ± 1.62
CMCS	158 ± 8.1	0.11 ± 0.05	−34.8 ± 3.3	N/A	N/A

N/A means Not applicable.

### 3.2 CMCS-20H Has Excellent Resistance Activity to Hydrolase Degradation

**Figure 2:**
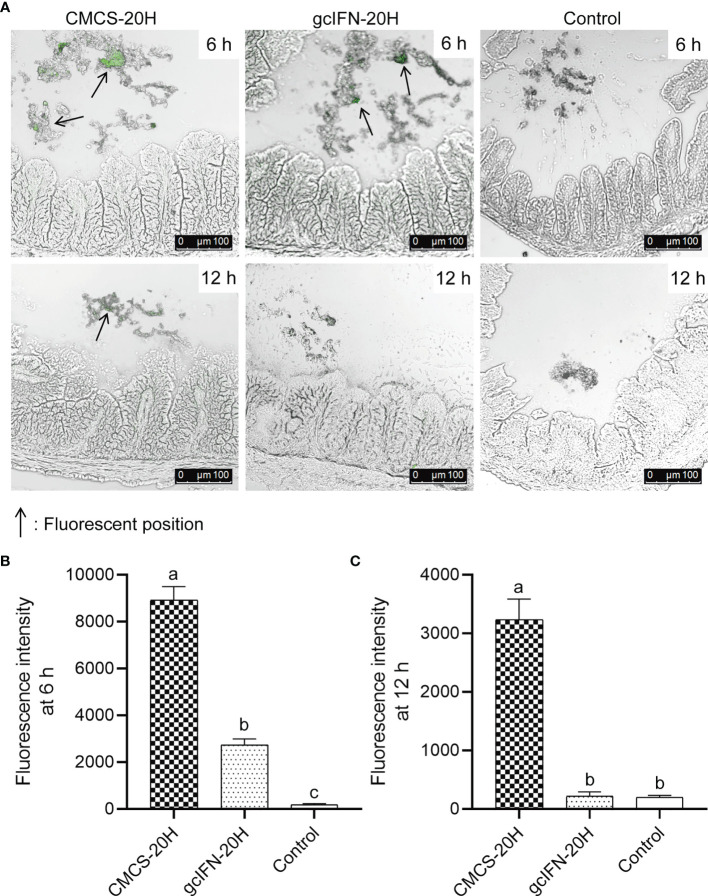
The antidegradation efficacy of CMCS-20H in intestine. **(A)** Fluorescence microscopic images of intestine section of grass carp. CMCS-20H and gcIFN-20H were poured into the foregut, and the hindgut was taken at 6 and 12 h to make frozen sections. The green fluorescence indicates the gcIFN-20H. **(B**, **C)** Fluorescence intensity analysis of intestine section at 6 and 12 h. Fluorescence intensity was analyzed by ImageJ software. Data are presented as means ± SD (*n* = 4). Different lowercase letters in each group (a, b, and c) denote significant variations suggested by the Kruskal-Wallis statistics followed by the Dunn’s multiple comparison (p < 0.05).

To explore the stability of CMCS-20H in intestine, CMCS-20H and gcIFN-20H were fed into the intestine of grass carp. By indirect immunofluorescence detection, the intestines of CMCS-20H group and gcIFN-20H group show obvious green fluorescence at 6 h. The green fluorescence in the CMCS-20H group is still be observed at 12 h, but the green fluorescence in the gcIFN-20H group disappeared (**Figure 2A**). Subsequently, the fluorescence intensity analysis of intestinal frozen sections showed that the fluorescence intensity in the CMCS-20H group was significantly higher than that in the gcIFN-20H group and control group at 6 and 12 h (**Figures 2B, C**). In addition, there was no significant difference in the fluorescence intensity between the gcIFN-20H group and control group at 12 h (**Figure 2C**). These results show that CMCS-20H possesses better stability and strongly resists degradation in intestine.

### 3.3 CMCS-20H Nanoparticles Effectively Establish Serum Immune Barrier Against Bacterial Infection

**Figure 3:**
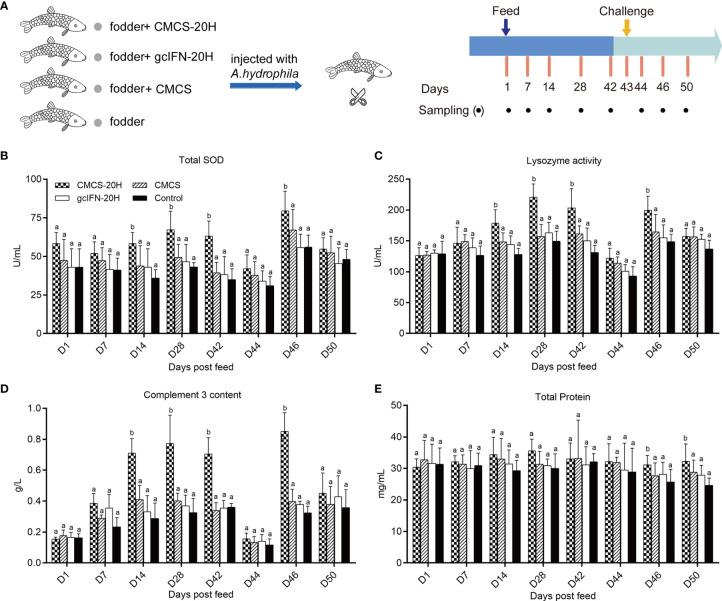
Serum biochemical indexes of innate immunity assay. **(A)** Oral administration, challenge, and sampling schedule. Fish in each group (*n* = 50) were feed with CMCS-20H+fodder, gcIFN-20H+fodder, CMCS+fodder, and fodder alone (control) and challenged with 100 µl *A. hydrophila* (2 × 10^5^ CFU/ml) on D43. **(B)** The assay of total superoxide dismutase (TSOD) activity. **(C)** The assay of lysozyme activity. **(D)** The assay of complement 3 content. **(E)** The assay of total protein content. Serum biochemical indexes were determined by the corresponding specific commercial kits (Nanjing Jiancheng Bioengineering Institute, Nanjing, China). Data are presented as means ± SD (*n* = 4). Different lowercase letters in each group (a and b) denote significant variations suggested by the Kruskal-Wallis statistics followed by the Dunn’s multiple comparison (p < 0.05).

The immune enhancement function of CMCS-20H was explored through *in vivo* experiments. The experimental flow and sampling time points are shown in **Figure 3A**. In the analyses of serum biochemical indexes before challenge, the activities of lysozyme and TSOD and complement C3 content were significantly higher than those in the gcIFN-20H, CMCS, and control groups on D14, D28, and D42 (**Figures 3B–D**). There was no significant difference of serum total protein concentration among the four groups (**Figure 3E**). After challenge, TSOD, lysozyme, and C3 in serum decreased on D44. They then increased and were significantly higher in the CMCS-20H group than those in the other three groups on D46 (**Figures 3B–D**). The total protein content in serum was significantly higher in the CMCS-20H group than that in the other three groups on D46 and D50 (**Figure 3E**). These data indicated that a beneficial serum immune barrier was induced by CMCS-20H to fight bacterial infections.

### 3.4 Oral Administration of CMCS-20H Nanoparticles Can Effectively Prevent Bacterial Infections by Improving Survival Rate and Immune Defense Capability, Alleviating Tissue Bacterial Load, Lesion, and Enteritis

**Figure 4:**
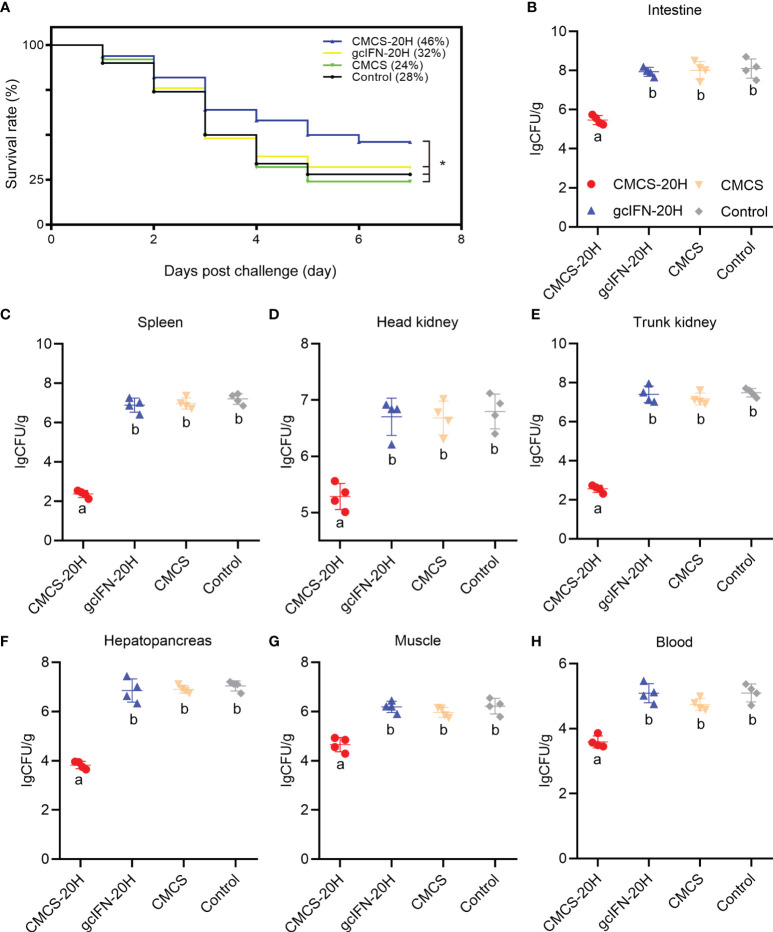
The survival rate and tissue bacterial load in grass carp post*-A. hydrophila* infection. **(A)** Survival rate was monitored and calculated for 7 days. **(B–H)** The content of *A. hydrophila* in each tissue (intestine, spleen, head kidney, trunk kidney, hepatopancreas, muscle, blood) in infected grass carp in each treatment group (*n* = 50). Samples were taken at 72 h postchallenge. Data are presented as means ± SD (*n* = 4). Different lowercase letters in each group (a and b) denote significant variations suggested by the Kruskal-Wallis statistics followed by the Dunn’s multiple comparison (p < 0.05). p values were calculated by Log-rank (Mantel-Cox) Test. * denotes significant variation (p < 0.05).

The survival rate in the CMCS-20H group (46%) was significantly higher than that in the gcIFN-20H (32%), CMCS (24%), and control (28%) groups. There was no significant difference in survival rate among the gcIFN-20H, CMCS, and control groups (**Figure 4A**). The CMCS-20H group dramatically reduced the content of *A. hydrophila* in grass carp tissues (intestine, spleen, head kidney, trunk kidney, hepatopancreas, muscle, and blood) by approximately 2–4 orders of magnitude (**Figures 4B–H**). These results indicated that CMCS-20H can efficiently prevent bacterial infections.

**Figure 5:**
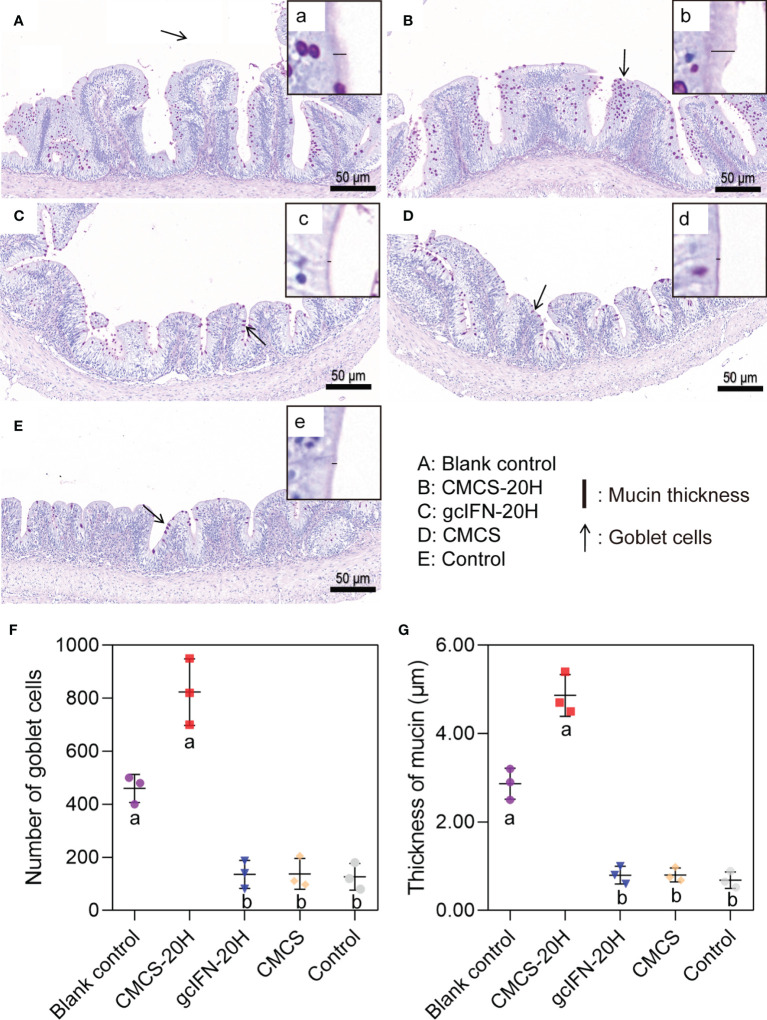
Mucus barrier investigations in grass carp intestine. **(A–E)** PAS staining of intestine section. The goblet cells are marked with black arrows. (a–e) Magnification of black box region to show the mucin thickness. The thickness of mucin was measured with a black line segment. **(F)** The number of goblet cells. Three fields (×20) were selected for goblet cell count. **(G)** The thickness measurement of mucin. Three fields (×70) were selected for goblet cell count. Samples were taken on D46. Different lowercase letters in each group (a and b) denote significant variations suggested by the Kruskal-Wallis statistics followed by the Dunn’s multiple comparison (p < 0.05).

The intestinal goblet cell number and mucin thickness were analyzed by PAS staining. Overall, the number of goblet cells in the CMCS-20H group increased, compared with blank control group. The number of goblet cells in groups gcIFN-20H, CMCS, and control decreased, compared with blank control group (**Figures 5A–E**). Compared with the blank control group, the mucins on intestinal villi in the CMCS-20H group increased, while the mucins in groups gcIFN-20H, CMCS, and control decreased (**Figures 5A–E**). Through the quantitative analysis of the number of goblet cells and mucin thickness, the number of goblet cells and mucin thickness in the CMCS-20H group were significantly higher than those in the other three groups. Compared with blank control group, the number of goblet cells and mucin thickness in the gcIFN-20H, CMCS, and control groups decreased significantly (**Figures 5F, G**).

**Figure 6:**
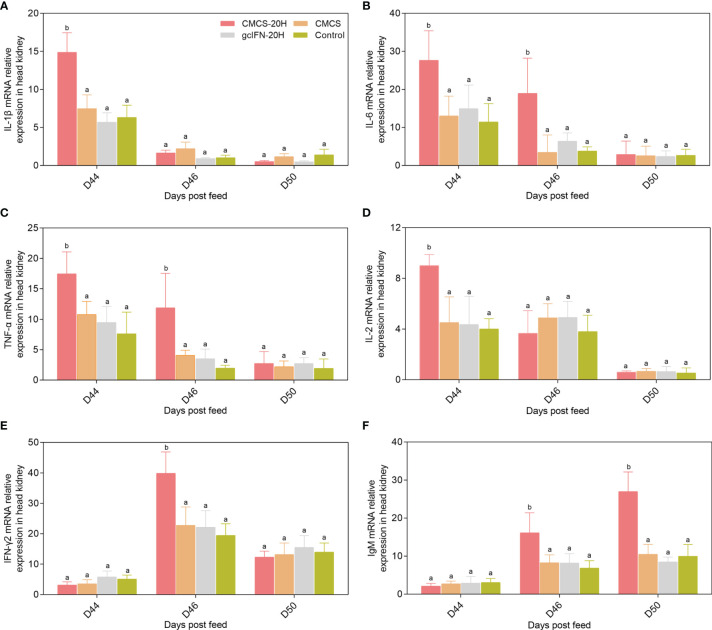
mRNA expression patterns of representative important immune regulation and effector genes in head kidney. **(A–F)** mRNA expressions of IL-1β, IL-6, TNF-α, IL-2, IFN-γ2, and IgM were determined by qRT-PCR. 18S rRNA gene was used as an internal control gene. Data are presented as means ± SD (*n* = 4). Different lowercase letters in each group (a and b) denote significant variations suggested by the Kruskal-Wallis statistics followed by the Dunn’s multiple comparison (p < 0.05).

mRNA expressions of representative immune genes including IL-1β, IL-6, TNF-α, IL-2, IFN-γ2, and IgM were examined by qRT-PCR in head kidney at different time points postfeeding and challenge. mRNA expressions of IL-1β, IL-6, TNF-α, and IL-2 in the CMCS-20H group were rapidly upregulated and significantly higher than those in other three groups on D44 (**Figures 6A–D**). mRNA expression of IFN-γ2 in the CMCS-20H group was significantly upregulated on D46 (**Figure 6E**). mRNA expression of IgM increased gradually and was significantly higher than the other three groups on D46 and D50 (**Figure 6F**).

**Figure 7:**
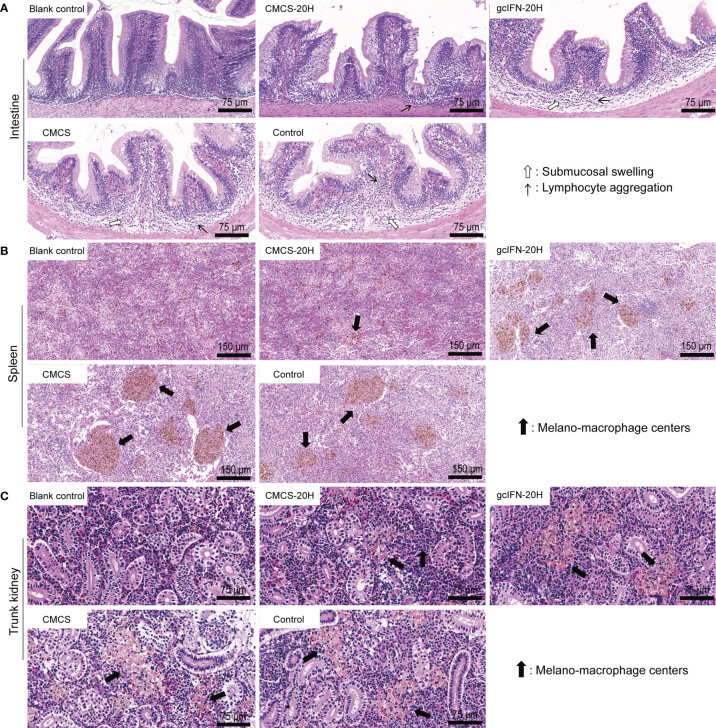
Tissue lesion assays of intestine, spleen, and trunk kidney postoral administration and challenge. On D46, the intestine, spleen and trunk kidney in grass carp were removed to make sections and stained by HE. Healthy grass carp tissues were used as blank control. **(A)** Histopathologic photographs of intestine. Submucosal swelling and lymphocyte aggregation were two main lesion signs in intestine. **(B)** Histopathologic photographs of spleen. Melano-macrophage centers were main lesion sign in spleen. **(C)** Histopathologic photographs of trunk kidney. Melano-macrophage centers were main lesion sign in trunk kidney.

To assess the extent of tissue lesion, intestine, spleen, and trunk kidney tissues were collected, fixed, and sliced for HE staining on D46. In intestinal sections, the gcIFN-20H, CMCS, and control groups showed obvious symptoms, such as submucosal swelling and lymphocyte aggregation. The CMCS-20H group had no symptom of submucosal swelling and the slight symptom of lymphocyte aggregation (**Figure 7A**). In spleen and trunk kidney sections, macrophage aggregation center was the most obvious lesion. The melano-macrophage centers in the gcIFN-20H, CMCS, and control groups were more serious than those in the CMCS-20H group (**Figures 7B, C**). Together, oral CMCS-20H can improve the survival rate in three ways: increasing the thickness of mucin and the number of goblet cells in the intestinal villi, improving mRNA expressions of the innate and adaptive immune genes, and reducing the tissue bacterial load and tissue lesion.

### 3.5 Oral Administration of CMCS-20H Can Maintain the Intestinal Microbiome Homeostasis in Bacterial Infection

**Figure 8:**
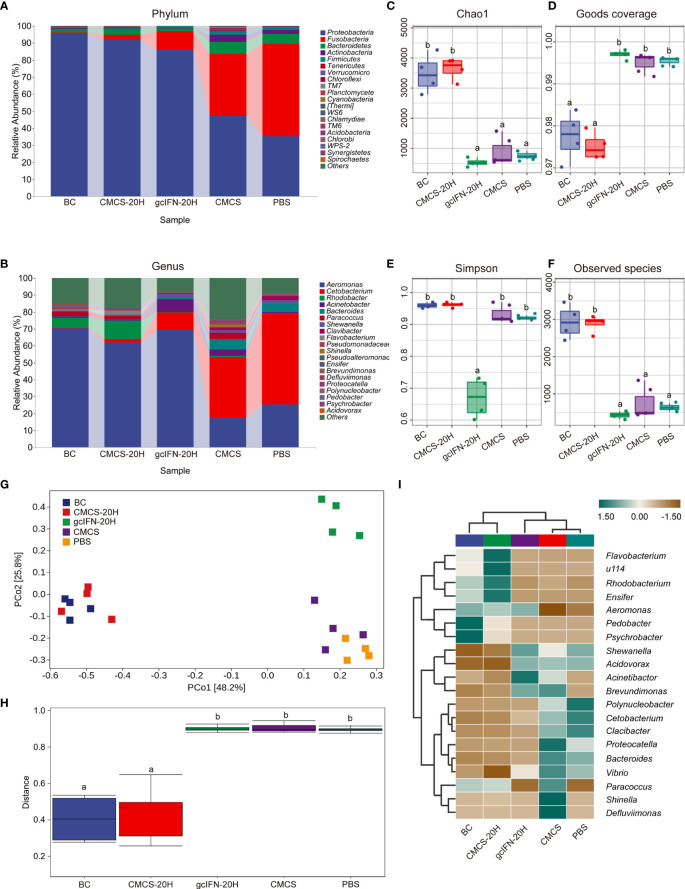
16S rRNA amplicon-sequencing analysis of intestinal microorganisms in grass carp. **(A, B)** Taxonomic composition analysis at phylum and genus levels. Grass carp fed with common fodder and unchallenged with *A*. *hydrophila* were used as blank control (BC). Each color bar represents one phylum and genus. The height of bar represents the phylum or genus relative abundance. “Others” includes the sum of different genera which are less than 1% in the sample. **(C–F)** Chao1, Goods coverage, Simpson, and Observed species were analyzed. **(G)** Principal coordinates (PCo) analysis. **(H)** Permutational multivariate analysis of variance (PERMANOVA) analysis. **(I)** Heatmap of hierarchy cluster results for the relative abundance of all samples at genus level. The sample information is shown along the horizontal axis. Annotations are shown along the vertical axis on the right. The genera clustering tree is shown along the vertical axis on the left. The sample clustering tree is shown above. The color represents the *Z*-score, which is the standardized relative abundance. Data are presented as means ± SD (*n* = 4). Different lowercase letters in each group (a and b) denote significant variations suggested by the Kruskal-Wallis statistics followed by the Dunn’s multiple comparison (p < 0.05).

16S rRNA gene sequencing method was used to evaluate the intestinal microorganisms in five groups. Abundance analysis of OTUs from five groups was carried out at levels of phylum and genus (**Figures 8A, B**). Compared with the gcIFN-20H, CMCS, and PBS groups, the relative abundance of *Proteobacteria* in the CMCS-20H group was highest and that of *Fusobacteria* was lowest at the phylum level. At the genus level, the relative richness of *Cetabacterium* in the CMCS-20H group was significantly lower than that of the gcIFN-20H, CMCS, and PBS groups. The intestinal microbiome richness in the CMCS-20H group was similar to that in the blank control group. The α-diversity (represented by Chao1, Goods coverage, Simpson, and Observed species indexes) of the gut microbiota was calculated in the five groups. According to Chao1 and Observed species indexes, the index richness in CMCS-20H and blank control groups were significantly higher than those in the gcIFN-20H, CMCS, and PBS groups (**Figures 8C, F**). The goods coverage value in the CMCS-20H and blank control groups were significantly lower than that in the gcIFN-20H, CMCS, and PBS groups (**Figure 8D**). The Simpson value in the gcIFN-20H group was significantly lower than that in the other four groups (**Figure 8E**). These results indicate that oral administration of CMCS-20H can effectively prevent the destruction of intestinal flora richness and maintain the stability of intestinal flora. PCoA analysis based on weighted UniFrac distances revealed that the bacterial composition in the CMCS-20H group was clearly segregated from that in the gcIFN-20H, CMCS, and PBS groups (**Figure 8G**). PERMANOVA was used to test the significant difference of sample distance among groups. After testing, the sample distance between the CMCS-20H group and the other three groups (gcIFN-20H, CMCS, and PBS) was significantly different (**Figure 8H**). In order to further compare the species composition differences between samples and display the species abundance distribution trend of each sample, we will use heatmap to display the species composition results. In species composition heatmap, we observed that the richness of *Flavobacterium*, *Rhodobacterium*, and *Ensifer* were the highest and that of *Acidovorax*, *Vibrio*, and *Shewanella* were the lowest in the CMCS-20H group (**Figure 8I**). These results indicate that oral CMCS-20H nanoparticles can effectively maintain the stability of intestinal microbiome in bacterial infection.

## 4 Discussion

Facing the detriment of fish bacterial diseases with high infectivity and high mortality, the development of immunopotentiator with low cost, no obvious toxicity, and excellent prevention effect remains a challenge and high priority (35). AMP family plays an important physiological role, such as bactericidal, immunoregulation, and others (32). Therefore, a great variety of AMPs have been developed as an immunopotentiator to prevent diseases (36, 37). In this study, a novel AMP gcIFN-20H was highly expressed in yeast and loaded on CMCS to form enzymatic hydrolysis-resistant CMCS-20H nanoparticles. The immunomodulatory function and the effect of preventing *A. hydrophila* infection by oral CMCS-20H were then explored on grass carp.

The methylotrophic yeast *P. pastoris* which has the advantages of simple operation, high yield, and low cost of production was extensively used in fundamental research and industry (23, 38). The gcIFN-20H DNA sequence was cloned into pPIC-9K plasmid between the α-factor secretion signal and the His-tag. Multicopy genes usually have a positive effect on yeast expression (22). In order to make the gene cassette carry a higher copy number of gcIFN-20H gene, a series concentration of G418 (0.2–0.8 mg/ml) was used to select the putative multicopy insertion transformants. In addition, the signal peptide can be naturally removed from the target protein without affecting the normal function of the target protein, and the target protein with His-tag can be easily separated from the culture medium (39). The results of SDS-PAGE analysis and WB assay showed that the secreted expressions of the target proteins were achieved by methanol induction in *P. pastoris*.

CMCS drug delivery nanotechnology provides a novel method to overcome the disadvantage of using peptide-based agents, especially in controlling the release and avoiding the digestion by enzymes (40, 41). The principle of CMCS binding to AMPs has confirmed that the negatively charged surface of CMCS and positively charged AMPs can attract each other through electrostatic interaction, and then form new nanoparticles by intermolecular forces (19). In this study, we successfully loaded AMP gcIFN-20H onto CMCS nanoparticles and obtained CMCS-20H nanoparticles. CMCS-20H has uniform particle size, good biocompatibility, and not obvious cytotoxicity. Previous studies have shown that free AMPs were readily degraded in tissues by hydrolases, causing the function of AMPs to be affected greatly (15). In the observation of intestinal frozen section, CMCS-20H can exist in the intestine for 12 h, and free gcIFN-20H can only exist for 6 h. This showed that CMCS-20H nanoparticles are more likely to function *in vivo* than free gcIFN-20H. CMCS-20H nanoparticles also have sustained release property, which can be released continuously for 20 h. Previous studies have shown that continuous release of AMPs can help nanoparticles function in the tissues (19, 42).

At present, CMCS drug delivery system has been widely used in the treatment of human diseases (18, 43), but few studies pay attention to the effect of loading AMPs on preventing fish diseases and improving immune response. In this study, the effect of oral CMCS-20H on serum biochemical indexes reflecting innate immunity was firstly investigated. Complement is a humoral factor of innate immunity and plays important roles in immune surveillance and clearance of invading pathogens. Complement 3 is a central molecule in the complement system whose activation is essential for all the important functions performed by this system (44). As having the same effect of lactoferrin (12), the activation of CMCS-20H to complement system can increase the content of C3 in serum. The main function of lysozyme in the process of disease resistance and immunity is to hydrolyze bacterial cell wall, leading to bacterial rupture and death, inducing and regulating the synthesis and secretion of other immune factors, so as to resist the invasion of foreign microorganisms (44). TSOD is an important antioxidant enzyme, which helps to remove the oxides produced by the body against pathogens (45). Previous studies showed that the resistance of grouper to bacterial and viral pathogens was related to the increase of TSOD and lysozyme activity (46). Compared with other experimental groups, the CMCS-20H group significantly increased the lysozyme activity in serum, indicating that oral administration of CMCS-20H helps grass carp establish a stronger innate immune barrier to resist the invasion of *A. hydrophila*. The highly active TSOD can help the body remove excessive oxides and help the body restore a stable state. After challenge, fish consume a large amount of C3, lysozyme, and TSOD in serum to eliminate *A. hydrophila*. Only biochemical indexes in the CMCS-20H group increase rapidly on D50, and the total protein concentration in the CMCS-20H group remains stable in the overall process. This indicates that the CMCS-20H group rapidly produce a beneficial serum innate immune response to fight bacterial infection, so as to keep the internal environment from damage. Noteworthy, innate immune response is not specific, leading to the strong innate immunity established by the oral administration of CMCS-20H to suffice to resist various types of pathogens (such as bacteria, viruses, and parasites). In addition, innate immunity can heighten protection upon reinfection with the same or unrelated pathogens (47).

The survival rate is the best reflection of the drug efficacy. In our previous study, the survival rate of the AMP hepcidin (60 mg/kg) group was 15% higher than that in the control group after injection of *Flavobacterium columnatus* (48). The survival rate of shrimp fed with AMP S100 (10 g/kg) after infection by *Harvey bacilli* was significantly higher than the control group (49). These AMPs are considered to be immunopotentiators with protective effects. In our experiment, the survival rate in the CMCS-20H group was significantly higher than that in the other three experimental groups. These results showed that CMCS-20H is an excellent immunopotentiator that can prevent bacterial infection.

Mucin is a highly glycosylated glycoprotein secreted by goblet cells (50). Mucin forms the skeleton of intestinal mucus layer, which is an important barrier against pathogens (51). The mucin in the CMCS-20H group help the body resist the invasion of *A. hydrophila*. This showed that CMCS-20H helps grass carp build a stable innate immune barrier, which is also one of the reasons to improve the survival rate. The CMCS-20H group significantly reduced the tissue bacterial load and the damage of *A. hydrophila* to visceral tissue. These effects are conducive to the body to produce beneficial adaptive and innate immune response to resist pathogenic infections (45). Therefore, it is necessary to explore the mechanism of oral CMCS-20H on innate and adaptive immunity. IL-1β, IL-6, and TNF-α are usually induced together to regulate immune responses and inflammation, which play an important role in regulating defensive and pathological innate immune responses (48). mRNA expressions of IL-1β, IL-6, and TNF-α in the CMCS-20H group were rapidly upregulated, suggesting that this treatment can better improve the inflammatory response. IL-2 promotes the proliferation and survival of activated T cells and has a strong upregulation effect on the effector cytokines (IFN-γ1, IFN-γ2, TNF-α, and IL-12) secreted by Th1 cells (52, 53). In addition, IL-2 is a cytokine necessary to activate B cells to synthesize immunoglobulin (52, 54). In this experiment, mRNA expressions of IL-2, IFN-γ2, and IgM in the CMCS-20H group were significantly upregulated at different time points, indicating that the cellular and humoral immunity in grass carp have been effectively improved.

Intestinal microorganisms form a symbiotic ecosystem, which helps maintain the steady balance in intestine and is essential to the health of humans and animals (14, 55). It is reported that intestinal inflammation will lead to changes in intestinal microorganisms, thus affecting the normal function of organisms (56). Through the sequencing of 16S rRNA gene by Illumina, we found that the relative abundance of *Proteobacteria* in the CMCS-20H group was highest and that of *Fusobacteria* was lowest. Previous research declared that the reduced *Proteobacteria* and elevated *Fusobacteria* may be related to the occurrence of intestinal inflammation (57, 58). α-Diversity in the exploration of diversity, Chao1, and observed values showed that the biodiversity in the CMCS-20H group was significantly higher than that in the gcIFN-20H, CMCS, and PBS groups. These results indicated that the intestinal inflammation in the CMCS-20H group is lower than the other challenged groups. In the principal coordinate analysis, we found that the distance between the CMCS-20H group and the other three groups (gcIFN-20H, CMCS, and PBS) was significantly different. The different distance between groups is caused by the difference of bacteria in each genus in the group (50, 55), in which different genera have been listed (*Aeromonas*, *Rhodobacter*, and *Cetabacterium*). We conclude that the intestinal inflammation in grass carp after oral administration of CMCS-20H is mild and the intestinal microbial richness is relatively stable, which is conducive to the normal function in grass carp.

## 5 Conclusion

CMCS-20H nanoparticles designed by us exhibit excellent features of sustained release and low toxicity, which can effectively prevent hydrolase degradation. Oral CMCS-20H treatment effectively enhances the activity and concentration of innate immune-related enzymes in serum. Oral CMCS-20H nanoparticles effectively reduce the mortality, tissue bacterial load, and tissue damage, significantly improve the innate and adaptive immune responses, and observably increase the thickness of mucin and the number of goblet cells in intestine in infected grass carp. In addition, the treatment of oral CMCS-20H improved the intestinal microbiome composition and microbial community balance. This study indicates that we have created a novel immunopotentiator and provide a method for the development of green AMPs for the prevention of fish diseases in practical large-scale aquaculture.

## Data Availability Statement

The datasets presented in this study can be found in online repositories. The names of the repository/repositories and accession number(s) can be found below: https://www.ncbi.nlm.nih.gov/genbank/, accession ID: PRJNA775959.

## Ethics Statement

All procedures of animal experiments were reviewed and approved by the Ethical Committee on Animal Research at Huazhong Agricultural University (ID Number: HZAUFI-2021-0004).

## Author Contributions

JS and XH conceived and designed the experiments and wrote the manuscript. XH, XX, ZW, and CY performed the experiments and analyzed the data. JS, XX, ZW, and CY revised the manuscript critically. All authors contributed to the article and approved the submitted version.

## Funding

This work was supported by the National Key Research and Development Program of China (2018YFD0900504) and National Natural Science Foundation of China (31873044).

## Conflict of Interest

The authors declare that the research was conducted in the absence of any commercial or financial relationships that could be construed as a potential conflict of interest.

## Publisher’s Note

All claims expressed in this article are solely those of the authors and do not necessarily represent those of their affiliated organizations, or those of the publisher, the editors and the reviewers. Any product that may be evaluated in this article, or claim that may be made by its manufacturer, is not guaranteed or endorsed by the publisher.

## References

[1] Gudding R Van Muiswinkel WB. A History of Fish Vaccination: Science-Based Disease Prevention in Aquaculture. Fish Shellfish Immunol (2013) 35:1683–8. doi: 10.1016/j.fsi.2013.09.031 24099805

[2] Golomazou E Malandrakis EE Panagiotaki P Karanis P. Cryptosporidium in Fish: Implications for Aquaculture and Beyond. Water Res (2021) 201:117357. doi: 10.1016/j.watres.2021.117357 34147739

[3] Khan SJ Osborn AM Eswara PJ. Effect of Sunlight on the Efficacy of Commercial Antibiotics Used in Agriculture. Front Microbiol (2021) 12:645175. doi: 10.3389/fmicb.2021.645175 34140934 PMC8203823

[4] Wu C Dai Y Yuan G Su J Liu X. Immunomodulatory Effects and Induction of Apoptosis by Different Molecular Weight Chitosan Oligosaccharides in Head Kidney Macrophages From Blunt Snout Bream (*Megalobrama Amblycephala*). Front Immunol (2019) 10:869. doi: 10.3389/fimmu.2019.00869 31156612 PMC6530513

[5] Mohd-Aris A Muhamad-Sofie MHN Zamri-Saad M Daud HM Ina-Salwany MY. Live Vaccines Against Bacterial Fish Diseases: A Review. Vet World (2019) 12:1806–15. doi: 10.14202/vetworld.2019.1806-1815 PMC6925058 32009760

[6] Hwang JY Kwon MG Seo JS Hwang SD Jeong JM Lee JH. Current Use and Management of Commercial Fish Vaccines in Korea. Fish Shellfish Immunol (2020) 102:20–7. doi: 10.1016/j.fsi.2020.04.004 32272258

[7] Su H Su J. Cyprinid Viral Diseases and Vaccine Development. Fish Shellfish Immunol (2018) 83:84–95. doi: 10.1016/j.fsi.2018.09.003 30195914 PMC7118463

[8] Embregts CW Forlenza M. Oral Vaccination of Fish: Lessons From Humans and Veterinary Species. Dev Comp Immunol (2016) 64:118–37. doi: 10.1016/j.dci.2016.03.024 27018298

[9] Bricknell I Dalmo RA. The Use of Immunostimulants in Fish Larval Aquaculture. Fish Shellfish Immunol (2005) 19:457–72. doi: 10.1016/j.fsi.2005.03.008 15890531

[10] Ouyang A Wang H Su J Liu X. Mannose Receptor Mediates the Activation of Chitooligosaccharides on Blunt Snout Bream (*Megalobrama Amblycephala*) Macrophages. Front Immunol (2021) 12:686846. doi: 10.3389/fimmu.2021.686846 34408745 PMC8365301

[11] Zasloff M. Antimicrobial Peptides of Multicellular Organisms: My Perspective. Adv Exp Med Biol (2019) 1117:3–6. doi: 10.1007/978-981-13-3588-4_1 30980349

[12] Costagliola G Nuzzi G Spada E Comberiati P Verduci E Peroni DG. Nutraceuticals in Viral Infections: An Overview of the Immunomodulating Properties. Nutrients (2021) 13:2410. doi: 10.3390/nu13072410 34371920 PMC8308811

[13] Ageitos JM Sanchez-Perez A Calo-Mata P Villa TG. Antimicrobial Peptides (AMPs): Ancient Compounds That Represent Novel Weapons in the Fight Against Bacteria. Biochem Pharmacol (2017) 133:117–38. doi: 10.1016/j.bcp.2016.09.018 27663838

[14] Wei X Zhang L Zhang R Koci M Si D Ahmad B. A Novel Cecropin-LL37 Hybrid Peptide Protects Mice Against EHEC Infection-Mediated Changes in Gut Microbiota, Intestinal Inflammation, and Impairment of Mucosal Barrier Functions. Front Immunol (2020) 11:1361. doi: 10.3389/fimmu.2020.01361 32695115 PMC7338479

[15] Lei R Hou J Chen Q Yuan W Cheng B Sun Y. Self-Assembling Myristoylated Human α-Defensin 5 as a Next-Generation Nanobiotics Potentiates Therapeutic Efficacy in Bacterial Infection. ACS Nano (2018) 12:5284–96. doi: 10.1021/acsnano.7b09109 29856606

[16] Moscoso-Mujica G Zavaleta AI Mujica A Arnao I Moscoso-Neira C Santos M. Antimicrobial Peptides Purified From Hydrolysates of Kanihua (*Chenopodium Pallidicaule* Aellen) Seed Protein Fractions. Food Chem (2021) 360:129951. doi: 10.1016/j.foodchem.2021.129951 33989882

[17] Tu Y Peng F Andre AA Men Y Srinivas M Wilson DA. Biodegradable Hybrid Stomatocyte Nanomotors for Drug Delivery. ACS Nano (2017) 11:1957–63. doi: 10.1021/acsnano.6b08079 PMC5348104 28187254

[18] Zhu X Yu Z Feng L Deng L Fang Z Liu Z. Chitosan-Based Nanoparticle Co-Delivery of Docetaxel and Curcumin Ameliorates Anti-Tumor Chemoimmunotherapy in Lung Cancer. Carbohydr Polym (2021) 268:118237. doi: 10.1016/j.carbpol.2021.118237 34127219

[19] Sun T Zhan B Zhang W Qin D Xia G Zhang H. Carboxymethyl Chitosan Nanoparticles Loaded With Bioactive Peptide OH-CATH30 Benefit Nonscar Wound Healing. Int J Nanomed (2018) 13:5771–86. doi: 10.2147/IJN.S156206 PMC6165789 30310280

[20] Nakahira Y Mizuno K Yamashita H Tsuchikura M Takeuchi K Shiina T. Mass Production of Virus-Like Particles Using Chloroplast Genetic Engineering for Highly Immunogenic Oral Vaccine Against Fish Disease. Front Plant Sci (2021) 12:717952. doi: 10.3389/fpls.2021.717952 34497627 PMC8419230

[21] Zhang W Zhu C Xiao F Liu X Xie A Chen F. pH-Controlled Release of Antigens Using Mesoporous Silica Nanoparticles Delivery System for Developing a Fish Oral Vaccine. Front Immunol (2021) 12:644396. doi: 10.3389/fimmu.2021.644396 33953716 PMC8089398

[22] Luo G Tian J Huang H Lei A. Improving Heterologous Expression of Porcine Follicle-Stimulating Hormone in *Pichia Pastoris* by Integrating Molecular Strategies and Culture Condition Optimization. Appl Microbiol Biotechnol (2018) 102:8867–82. doi: 10.1007/s00253-018-9260-6 30136206

[23] Li J Xie X Cai J Wang H Yang J. Enhanced Secretory Expression and Surface Display Level of *Bombyx Mori* Acetylcholinesterase 2 by *Pichia Pastoris* Based on Codon Optimization Strategy for Pesticides Setection. Appl Biochem Biotechnol (2021) 193:3321–35. doi: 10.1007/s12010-021-03597-7 34160750

[24] Yan G Shu M Shen W Ma L Zhai C Wang Y. Heterologous Expression of Nattokinase From *B. Subtilis* Natto Using *Pichia Pastoris* GS115 and Assessment of its Thrombolytic Activity. BMC Biotechnol (2021) 21:49. doi: 10.1186/s12896-021-00708-4 34372833 PMC8353737

[25] Schroers V van der Marel M Neuhaus H Steinhagen D. Changes of Intestinal Mucus Glycoproteins After Peroral Application of *Aeromonas Hydrophila* to Common Carp (*Cyprinus Carpio*). Aquaculture (2009) 288:184–9. doi: 10.1016/j.aquaculture.2008.12.013

[26] Song X Zhao J Bo Y Liu Z Wu K Gong C. *Aeromonas Hydrophila* Induces Intestinal Inflammation in Grass Carp (*Ctenopharyngodon Idella*): An Experimental Model. Aquaculture (2014) 434:171–8. doi: 10.1016/j.aquaculture.2014.08.015

[27] Sughra F Rahman MH Abbas F Altaf I. Evaluation of Three Alum-Precipitated *Aeromonas Hydrophila* Vaccines Administered to *Labeo Rohita*, *Cirrhinus Mrigala* and *Ctenopharyngodon Idella*: Immunokinetics, Immersion Challenge and Histopathology. Braz J Biol (2021) 83:e249913. doi: 10.1590/1519-6984.249913 34550293

[28] Xiao X Zhu W Zhang Y Liao Z Wu C Yang C. Broad-Spectrum Robust Direct Bactericidal Activity of Fish Ifnφ1 Reveals an Antimicrobial Peptide-Like Function for Type I IFNs in Vertebrates. J Immunol (2021) 206:1337–47. doi: 10.4049/jimmunol.2000680 33568398

[29] Wang J Kong M Zhou Z Yan D Yu X Cheng X. Mechanism of Surface Charge Triggered Intestinal Epithelial Tight Junction Opening Upon Chitosan Nanoparticles for Insulin Oral Delivery. Carbohydr Polym (2017) 157:596–602. doi: 10.1016/j.carbpol.2016.10.021 27987967

[30] Gao P Xia G Bao Z Feng C Cheng X Kong M. Chitosan Based Nanoparticles as Protein Carriers for Efficient Oral Antigen Delivery. Int J Biol Macromol (2016) 91:716–23. doi: 10.1016/j.ijbiomac.2016.06.015 27287772

[31] Dai C Basilico P Cremona TP Collins P Moser B Benarafa C. CXCL14 Displays Antimicrobial Activity Against Respiratory Tract Bacteria and Contributes to Clearance of *Streptococcus Pneumoniae* Pulmonary Infection. J Immunol (2015) 194:5980–9. doi: 10.4049/jimmunol.1402634 25964486

[32] Mei D Guo X Wang Y Huang X Guo L Zou P. PEGylated Graphene Oxide Carried OH-CATH30 to Accelerate the Healing of Infected Skin Wounds. Int J Nanomed (2021) 16:4769–80. doi: 10.2147/IJN.S304702 PMC8286112 34285482

[33] Hu Y Kurobe T Liu X Zhang YA Su J Yuan G. Hamp Type-1 Promotes Antimicrobial Defense *via* Direct Microbial Killing and Regulating Iron Metabolism in Grass Carp (*Ctenopharyngodon Idella*). Biomolecules (2020) 10:825. doi: 10.3390/biom10060825 PMC7356000 32481513

[34] Zhang Y Xiao X Hu Y Liao Z Zhu W Jiang R. CXCL20a, a Teleost-Specific Chemokine That Orchestrates Direct Bactericidal, Chemotactic, and Phagocytosis-Killing-Promoting Functions, Contributes to Clearance of Bacterial Infections. J Immunol (2021) 207:1911–25. doi: 10.4049/jimmunol.2100300 34462313

[35] Miccoli A Saraceni PR Scapigliati G. Vaccines and Immune Protection of Principal Mediterranean Marine Fish Species. Fish Shellfish Immunol (2019) 94:800–9. doi: 10.1016/j.fsi.2019.09.065 31580938

[36] Petkovic M Mouritzen MV Mojsoska B Jenssen H. Immunomodulatory Properties of Host Defence Peptides in Skin Wound Healing. Biomolecules (2021) 11:952. doi: 10.3390/biom11070952 34203393 PMC8301823

[37] Ramuta TZ Sket T Starcic Erjavec M Kreft ME. Antimicrobial Activity of Human Fetal Membranes: From Biological Function to Clinical Use. Front Bioeng Biotechnol (2021) 9:691522. doi: 10.3389/fbioe.2021.691522 34136474 PMC8201995

[38] Macauley-Patrick S Fazenda ML McNeil B Harvey LM. Heterologous Protein Production Using the *Pichia Pastoris* Expression System. Yeast (2005) 22:249–70. doi: 10.1002/yea.1208 15704221

[39] Zhou Y Jiang N Ma J Fan Y Zhang L Xu J. Protective Immunity in Gibel Carp, *Carassius Gibelio* of the Truncated Proteins of Cyprinid Herpesvirus 2 Expressed in *Pichia Pastoris* . Fish Shellfish Immunol (2015) 47:1024–31. doi: 10.1016/j.fsi.2015.11.012 26564473

[40] Wang Y Ma J Qiu T Tang M Zhang X Dong W. *In Vitro* and *In Vivo* Combinatorial Anticancer Effects of Oxaliplatin- and Resveratrol-Loaded N,O-Carboxymethyl Chitosan Nanoparticles Against Colorectal Cancer. Eur J Pharm Sci (2021) 163:105864. doi: 10.1016/j.ejps.2021.105864 33965502

[41] Narayanan D Jayakumar R Chennazhi KP. Versatile Carboxymethyl Chitin and Chitosan Nanomaterials: A Review. Wiley Interdiscip Rev Nanomed Nanobiotechnol (2014) 6:574–98. doi: 10.1002/wnan.1301 25266740

[42] Zou P Lee WH Gao Z Qin D Wang Y Liu J. Wound Dressing From Polyvinyl Alcohol/Chitosan Electrospun Fiber Membrane Loaded With OH-CATH30 Nanoparticles. Carbohydr Polym (2020) 232:115786. doi: 10.1016/j.carbpol.2019.115786 31952594

[43] Nguyen CT Nguyen TT Nguyen TT Nguyen PPT Nguyen AD Tran LT. Preparation and *In Vitro* Evaluation of FGF-2 Incorporated Carboxymethyl Chitosan Nanoparticles. Carbohydr Polym (2017) 173:114–20. doi: 10.1016/j.carbpol.2017.05.080 28732849

[44] Yan Y Huo X Ai T Su J. β-Glucan and Anisodamine can Enhance the Immersion Immune Efficacy of Inactivated Cyprinid Herpesvirus 2 Vaccine in *Carassius Auratus Gibelio* . Fish Shellfish Immunol (2020) 98:285–95. doi: 10.1016/j.fsi.2020.01.025 31962149

[45] Zhu W Zhang Y Zhang J Yuan G Liu X Ai T. Astragalus Polysaccharides, Chitosan and Poly(I:C) Obviously Enhance Inactivated *Edwardsiella Ictaluri* Vaccine Potency in Yellow Catfish *Pelteobagrus Fulvidraco* . Fish Shellfish Immunol (2019) 87:379–85. doi: 10.1016/j.fsi.2019.01.033 30690155

[46] Chiu S-T Tsai R-T Hsu J-P Liu C-H Cheng W. Dietary Sodium Alginate Administration to Enhance the non-Specific Immune Responses, and Disease Resistance of the Juvenile Grouper *Epinephelus Fuscoguttatus* . Aquaculture (2008) 277:66–72. doi: 10.1016/j.aquaculture.2008.01.032

[47] Moorlag S Khan N Novakovic B Kaufmann E Jansen T van Crevel R. β-Glucan Induces Protective Trained Immunity Against *Mycobacterium Tuberculosis* Infection: A Key Role for IL-1. Cell Rep (2020) 31:107634. doi: 10.1016/j.celrep.2020.107634 32433977 PMC7242907

[48] Chen T Zhou J Qu Z Zou Q Liu X Su J. Administration of Dietary Recombinant Hepcidin on Grass Carp (*Ctenopharyngodon Idella*) Against *Flavobacterium Columnare* Infection Under Cage Aquaculture Conditions. Fish Shellfish Immunol (2020) 99:27–34. doi: 10.1016/j.fsi.2020.01.042 32001352

[49] Wu S Zhang F Huang Z Liu H Xie C Zhang J. Effects of the Antimicrobial Peptide Cecropin AD on Performance and Intestinal Health in Weaned Piglets Challenged With *Escherichia Coli* . Peptides (2012) 35:225–30. doi: 10.1016/j.peptides.2012.03.030 22490448

[50] Yi H Hu W Chen S Lu Z Wang Y. Cathelicidin-WA Improves Intestinal Epithelial Barrier Function and Enhances Host Defense Against Enterohemorrhagic *Escherichia Coli* O157:H7 Infection. J Immunol (2017) 198:1696–705. doi: 10.4049/jimmunol.1601221 28062699

[51] Gallo A Passaro G Gasbarrini A Landolfi R Montalto M. Modulation of Microbiota as Treatment for Intestinal Inflammatory Disorders: An Uptodate. World J Gastroenterol (2016) 22:7186–202. doi: 10.3748/wjg.v22.i32.7186 PMC4997632 27621567

[52] Wang T Hu Y Wangkahart E Liu F Wang A Zahran E. Interleukin (IL)-2 is a Key Regulator of T Helper 1 and T Helper 2 Cytokine Expression in Fish: Functional Characterization of Two Divergent IL2 Paralogs in Salmonids. Front Immunol (2018) 9:1683. doi: 10.3389/fimmu.2018.01683 30093902 PMC6070626

[53] Diaz-Rosales P Bird S Wang TH Fujiki K Davidson WS Zou J. Rainbow Trout Interleukin-2: Cloning, Expression and Bioactivity Analysis. Fish Shellfish Immunol (2009) 27:414–22. doi: 10.1016/j.fsi.2009.06.008 19540920

[54] Yu TK Caudell EG Smid C Grimm EA. IL-2 Activation of NK Cells: Involvement of MKK1/2/ERK But Not P38 Kinase Pathway. J Immunol (2000) 164:6244–51. doi: 10.4049/jimmunol.164.12.6244 10843677

[55] Cao S-L Guo J-J Zhao W-P Yang W-F Zhang S-L Tao H-Z. Impacts of Oral *Vibrio Mimicus* Double-Targeted DNA Vaccine on the Gut Microbiota in Grass Carps (*Ctenopharyngodon Idella*) and Correlations With Intestinal Mucosa Innate Immunity. Aquaculture (2021) 533:736201. doi: 10.1016/j.aquaculture.2020.736201

[56] Mima K Ogino S Nakagawa S Sawayama H Kinoshita K Krashima R. The Role of Intestinal Bacteria in the Development and Progression of Gastrointestinal Tract Neoplasms. Surg Oncol (2017) 26:368–76. doi: 10.1016/j.suronc.2017.07.011 PMC5726560 29113654

[57] Shin NR Whon TW Bae JW. *Proteobacteria*: Microbial Signature of Dysbiosis in Gut Microbiota. Trends Biotechnol (2015) 33:496–503. doi: 10.1016/j.tibtech.2015.06.011 26210164

[58] Lavelle A Lennon G O’Sullivan O Docherty N Balfe A Maguire A. Spatial Variation of the Colonic Microbiota in Patients With Ulcerative Colitis and Control Volunteers. Gut (2015) 64:1553–61. doi: 10.1136/gutjnl-2014-307873 PMC4602252 25596182

