# Supplementary material for: Oral Administration of Nanopeptide CMCS-20H Conspicuously Boosts Immunity and Precautionary Effect Against Bacterial Infection in Fish
Source: Front Immunol. 2022 Jan 11;12:811616. doi: 10.3389/fimmu.2021.811616 (PMC8786714; doi:10.3389/fimmu.2021.811616)
Supplement: Supplementary file 1 [file DataSheet_1.docx]

# Supplementary Figures

***
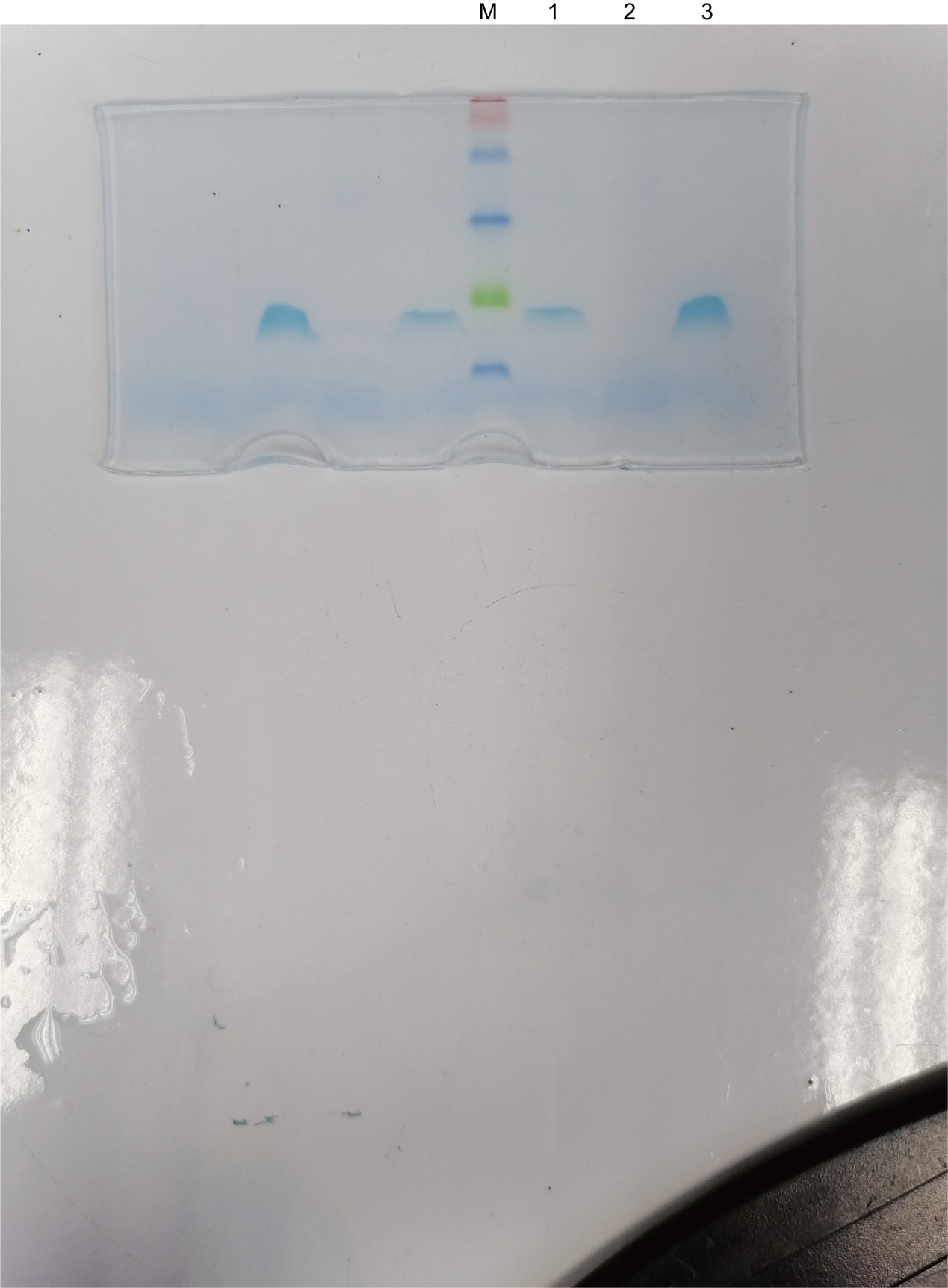
***

This SDS-PAGE assay result is the original image of Fig 1A. Lane M: protein marker; Lane 1: the fermentation supernatant of gcIFN-20H; Lane 2: the fermentation supernatant of blank vector as negative control; Lane 3: purified gcIFN-20H.

*
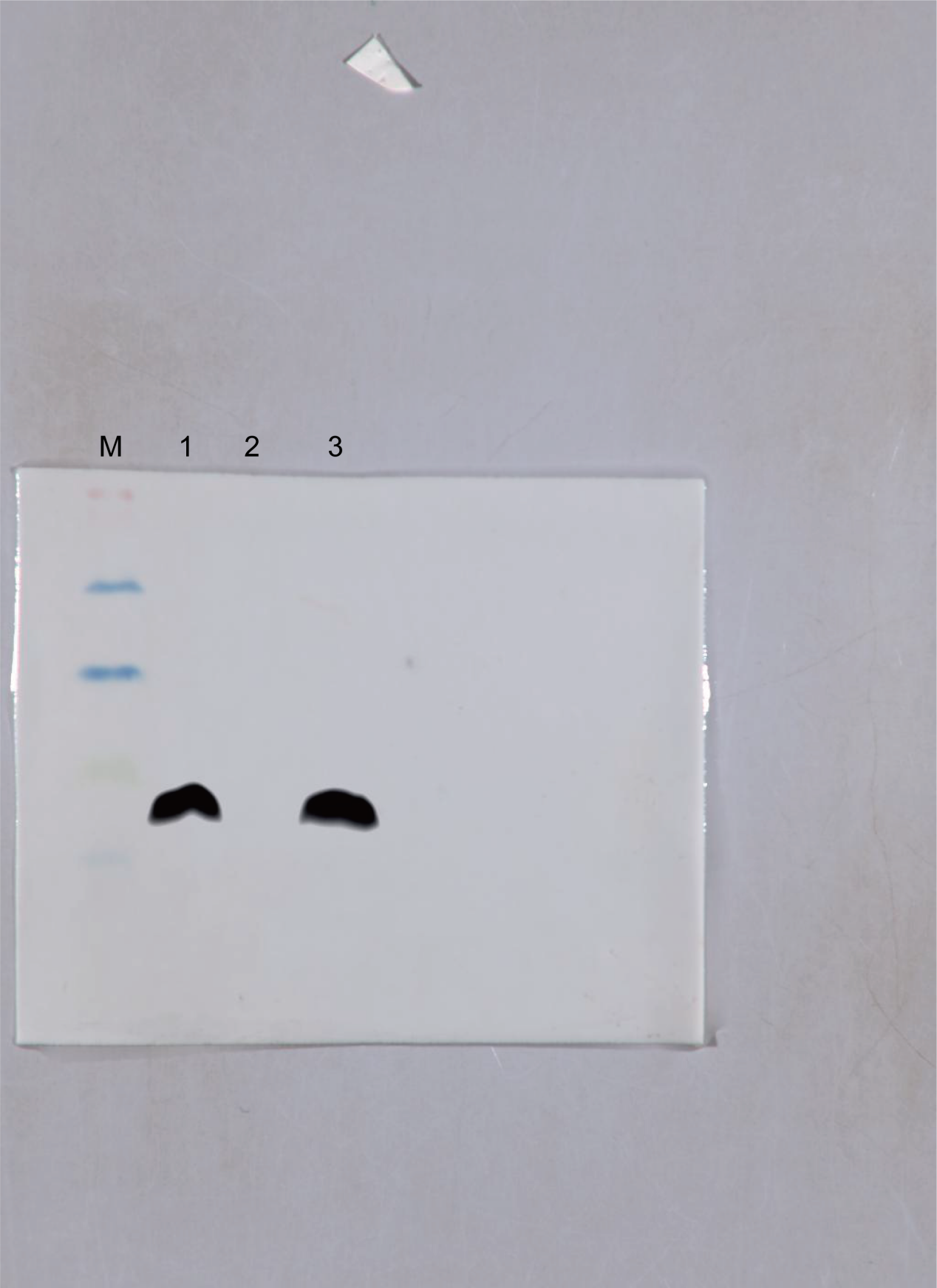
*

This WB assay result is the original image of Fig 1B. Lane M: protein marker; Lane 1: the fermentation supernatant of gcIFN-20H; Lane 2: the fermentation supernatant of blank vector as negative control; Lane 3: purified gcIFN-20H.
