# Supplementary material for: Oral Administration of Nanopeptide CMCS-20H Conspicuously Boosts Immunity and Precautionary Effect Against Bacterial Infection in Fish
Source: Front Immunol. 2022 Jan 11;12:811616. doi: 10.3389/fimmu.2021.811616 (PMC8786714; doi:10.3389/fimmu.2021.811616)
Supplement: Supplementary file 2 [file Table_1.docx]

# Supplementary Tables

## Table S1 Primer sequences for qRT-PCR assays in this study.

| Gene name | Primer direction | Primer sequence (5’-3’) | Size (bp) |
| --- | --- | --- | --- |
| IL-1β | Forward | AAGTTCCCGCTTTGGAGAGTA | 126 |
|  | Reverse | GCCACATACCAGTCGTTCAGT |  |
| IL-2 | Forward | CATCAAGCTCACCCCGAAAC | 93 |
|  | Reverse | GTAGTTGCACCAGACGTTCC |  |
| IFN-γ2 | Forward | GCCTGGACACAGCTACAAAAG | 102 |
|  | Reverse | ATGTCTTGAGCTCTGCACTCT |  |
| IL-6 | Forward | AGGAAGGCTCCAGGGTTACA | 116 |
|  | Reverse | GCTGAGATGCGGACGTCTTA |  |
| IgM | Forward | TGGAGCAACGGCACAGTATT | 131 |
|  | Reverse | TCTGGGGGTGCTAACAGGTA |  |
| TNF-α | Forward | CCAGCTCTTCCCAAACCAGT | 126 |
|  | Reverse | CCATCATCCTTCGCCCATGA |  |
| 18S rRNA | Forward | ATTTCCGACACGGAGAGG | 90 |
|  | Reverse | CATGGGTTTAGGATACGCTC |  |
